# Developing a Machine Learning–Based System for Nutrient Profiling of Pakistani Cuisine

**DOI:** 10.3390/nu18193173

**Published:** 2026-09-26

**Authors:** Alishba Umer Farooq, Fiza Khan, Huda Ijaz, Muhammad Usman, Nimra Saleem, Shuja ur Rehman Baig, Muhammad Farooq, Syed Mustafa Ali

**Affiliations:** 1 Faculty of Computing and Information Technology, The University of Punjab, Lahore P.O. Box 54590, Pakistan; alishbaumer7800@gmail.com (A.U.F.); fizakhan1057@gmail.com (F.K.); hudaejaz823@gmail.com (H.I.); shuja@pucit.edu.pk (S.u.R.B.); mfarooq@pucit.edu.pk (M.F.); 2 Pakistan Scientific and Technological Information Center, Islamabad P.O. Box 45320, Pakistan; usmiusman@gmail.com; 3 APPNA Institute of Public Health, Jinnah Sindh Medical University, Karachi P.O. Box 75510, Pakistan; 4 Division of Informatics, Imaging and Data Science, Manchester Academic Health Science Centre, University of Manchester, Manchester M13 9PL, UK

**Keywords:** computer vision, machine learning, nutrition assessment, public health, patient generated health data

## Abstract

Background: Chronic diseases such as diabetes and heart disease are rising in South Asia, with poor diet a major contributor. Most dietary apps are designed for Western foods and struggle with culturally diverse dishes such as Pakistani cuisine. Objective: This study aimed to build a machine learning-based system that can recognise Pakistani dishes, estimate portion sizes, and calculate their nutritional value. Methods: We developed a multi-stage automated dietary assessment pipeline for Pakistani cuisine using a multi-source dataset covering 83 dish classes across 10 categories. Following data cleaning, class balancing, and targeted augmentation, deep learning architectures were evaluated for food classification and segmentation. ResNet-50, MobileNetV2, and YOLOv8 were used for food recognition, while YOLOv8-Seg and Mask R-CNN enabled pixel-level segmentation for portion and nutrient estimation. All models were optimized using transfer learning and evaluated using standard classification and mask-quality metrics, followed by mobile application usability testing. Results: A multi-source dataset of ten categories and 83 Pakistani dishes was used to train and compare deep learning models for food classification and segmentation. Portion size was estimated using a segmentation- and depth-based approach without a reference object, while nutritional values were derived from standardised recipes and established food databases. The models were integrated into a mobile application for usability testing. YOLO-based models achieved the best balance of accuracy and speed for real-time classification and segmentation. Portion estimates were reasonably close to measured values, and usability testing showed the app was easy to use, with minor improvements suggested. Conclusions: Overall, this work demonstrates the feasibility of an AI-based dietary assessment system adapted to Pakistani cuisine, with future work focused on reducing processing demands and improving depth-based portion estimation.

## 1. Introduction

The global prevalence of long-term conditions such as diabetes, hypertension, cardiovascular diseases and chronic pain is rising rapidly, particularly in low- and middle-income countries. In these countries, healthcare infrastructure is over-burdened and fragmented and rapid urbanization, and lifestyle changes have worsened health outcomes [1]. Over three-quarters of deaths from long-term conditions occur in these regions [2], and South Asian and Black African communities living in high-income countries show similar trends due to genetic risk, lifestyle factors, and unequal healthcare access [3,4]. Among the lifestyle factors, poor dietary habits contribute significantly to the development and progression of long-term conditions, underscoring the need for scalable strategies to promote healthy eating and nutritional awareness [5].

Dietary assessment tools are mainstay of the current strategies to monitor and improve dietary intake [6]. Traditional methods, such as food diaries and 24-h recalls, can support behavior change but are often burdensome, time-consuming, and prone to inaccuracy [7,8]. They also fail to capture culturally specific foods and mixed dishes, reducing accuracy in nutrient and calorie estimation, particularly for South Asian diets [9,10,11]. This challenge is particularly relevant to Pakistani cuisine, which includes diverse mixed dishes with substantial variation in ingredients, preparation methods, and portion sizes.

Recent research has explored AI-driven approaches for automated dietary assessment, offering a promising alternative for automated dietary tracking via consumer devices [12]. These systems are well established for Western diets, with commercial nutrition apps widely being evaluated [13]. However, research on South Asian cuisine remains largely at the prototype stage and does not capture food and culinary diversity among South Asian countries [14]. Machine learning-based approaches offer the potential to address these limitations through automated food recognition, segmentation, portion estimation, and nutrient profiling.

Despite the potential of AI-based dietary assessment, many AI-based systems misidentify mixed or traditional South Asian (i.e., Pakistani) dishes, providing inaccurate nutrient estimates and culturally irrelevant advice [15,16]. Existing state-of-the-art systems (e.g., goFOOD, FoodAI) rely on structured meals and Western datasets. This limits their performance in mixed-dish South Asian meals, where visual overlap between ingredients and high intra-dish variability are common. Specific technical limitations include the lack of diverse annotated datasets [17], difficulties in segmenting mixed foods [18], and challenges in estimating portion sizes for complex dishes such as curries, rice meals, and flatbreads without physical reference objects [19]. An effective system for Pakistani cuisine therefore requires not only accurate food recognition and portion estimation but also reliable nutrient profiling based on culturally relevant food information.

These challenges highlight the need for an end-to-end, culturally adaptive dietary assessment system that can accurately recognize, segment, and estimate portions of complex mixed dishes in Pakistani cuisine. Therefore, the aim of this study was to develop a machine learning-based system for accurate nutrient profiling of Pakistani cuisine. We achieved this by following objectives:

1. Set up a culturally specific, multi-sourced labeled dataset of Pakistani cuisine;
2. Develop a hybrid segmentation and monocular depth-based portion estimation pipeline that does not require physical reference objects;
3. Develop and test an end-to-end machine learning system integrating image classification, instance segmentation, monocular depth estimation, and nutritional computation into a unified mobile pipeline;
4. Conduct preliminary validation of both system accuracy and real-world usability.

## 2. Methodology

This section describes the methodology adopted to develop and evaluate the proposed AI-based dietary assessment system. It covers dataset acquisition and preprocessing, model selection, training and evaluation, segmentation-based portion and nutritional estimation, integration of the trained models into the mobile application, and usability testing. The complete workflow is presented to provide a systematic account of the development and evaluation of the proposed system.

### 2.1. Overview of the Proposed System

This study proposes a multi-stage pipeline for automated dietary assessment of Pakistani cuisine, integrating food recognition, segmentation, portion estimation, and nutritional profiling into a unified framework. The pipeline is designed to process food images from initial data preparation and model development through portion and nutrient estimation, and ultimately support real-time dietary analysis through a smartphone application. The proposed pipeline consists of the following stages, which is also graphically represented in Figure 1:

(i) multi-source dataset construction from publicly available images, including cafeteria captures and user-contributed photographs;
(ii) data cleaning, annotation, and class balancing through targeted augmentation;
(iii) training and evaluation of deep learning models for food recognition, segmentation, and depth-based portion estimation;
(iv) integration of deep learning models into a nutrient profiling module based on standardized food composition data; and
(v) deployment as a smartphone application for real-time dietary analysis.

### 2.2. Dataset Acquisition

A multi-source image acquisition strategy was adopted, including web scraping and YouTube thumbnails, crowd-sourced contributions, and publicly available external datasets (see Supplementary Materials for details—dataset acquisition). Though image sources varied in visual context, intent, and presentation, this multi-source approach was adopted to train the model on both real-world user-captured conditions and digital media representations, thereby improving generalisability across the range of image types. Using this approach, we constructed a balanced dataset of Pakistani food images organized into ten semantically distinct categories, capturing the diversity of Pakistani cuisine, as summarized in Table 1. The dataset is further divided into 83 fine-grained dish classes, forming the basis for dataset construction, annotation, and subsequent model training and evaluation. A stratified split of 70% for training, 15% for validation, and 15% for testing was applied to preserve class distribution, better generalization or un-biased evaluation.

### 2.3. Data Preprocessing and Quality Assurance

The dataset was preprocessed to ensure data quality using established practices in food recognition systems [20], including removal of duplicate and low-quality images (e.g., blurred or heavily occluded samples), as well as resizing and normalising model input requirements.

Prior to the model training, class labels assigned during data acquisition were manually verified through duplicate verification process conducted independently by multiple researchers or authors. Instances of mislabelling were identified and corrected during this process, while mislabelling was predominantly attributable to visual similarity between classes (e.g., dishes with comparable color, texture, or presentation).

To improve model robustness and generalisability, data augmentation techniques were applied exclusively to the training set. These transformations included random rotation within ±15°, horizontal flipping with a probability of 0.5, brightness and contrast adjustments within ±20%, Gaussian noise injection, and random scaling within a range of 0.8–1.2. Augmentation parameters were selected conservatively to preserve the structural characteristics of food items. No augmentation was applied to validation or test sets to prevent data leakage.

To ensure dataset reliability and support robust model training, a series of validation and quality assurance measures were implemented. The distribution of images across the ten food categories was analyzed to identify class imbalance [21]. Initial analysis revealed that certain categories, such as rice dishes and meat curries, were overrepresented, while others, including desserts and lentil-based dishes, were underrepresented. To address this imbalance, a combination of controlled oversampling of minority classes and targeted data augmentation was applied.

A combination of controlled oversampling of minority classes and targeted data augmentation was applied by maintaining the category distribution within a controlled range (8–15% of the dataset). Oversampling helped reduce class bias for fair model training thereby improving generalisation.

### 2.4. Model Selection and Architecture

To address the variability in food appearance, texture, and presentation in Pakistani cuisine, multiple deep learning architectures were selected and evaluated based on their complementary strengths. The selection criteria focused on three key aspects, including classification accuracy, computational efficiency, and suitability for mobile deployment.

For image classification, ResNet-50 [22] was employed due to its residual learning framework, which enables deep feature extraction while mitigating vanishing gradient issues. Transfer learning using ImageNet-pretrained weights [23] facilitated adaptation to domain-specific food features. MobileNetV2 [24] was selected as a lightweight alternative optimised for mobile environments, utilising depth wise, separable convolutions to reduce computational complexity while maintaining competitive performance.

For real-time classification, YOLOv8-s (small) classification variant [25] was included due to its low latency and strong performance in single-stage inference scenarios.

For segmentation tasks, YOLOv8-Seg (small variant) [25] was used to provide pixel-level localization of food items, which is essential for portion estimation. Additionally, Mask R-CNN [26] was employed as a benchmark segmentation model, leveraging region-based detection and a parallel mask prediction branch for precise boundary extraction. Transfer learning with COCO-pretrained weights [27] was applied to improve segmentation performance.

### 2.5. Model Training and Evaluation Matrix

All models were trained for 50 epochs using the AdamW optimizer [27] with a learning rate of 0.05. Batch sizes were set to 32 for classification models (ResNet-50, MobileNetV2, YOLOv8-s) and 4 for segmentation models (YOLOv8-Seg, Mask R-CNN), reflecting the higher memory demands of segmentation training. Training was conducted on NVIDIA T4 GPUs accessed via Google Colab and Kaggle. For classification, ResNet-50 and MobileNetV2 were initialized with ImageNet-pretrained weights [28], and the YOLOv8-s classification variant was initialized with weights pretrained by Ultralytics, prior to fine-tuning on our dataset. For segmentation, YOLOv8-Seg and Mask R-CNN were initialized with COCO-pretrained weights [27], consistent with standard transfer learning practice for instance segmentation. No additional hyperparameter tuning was performed beyond the settings described above.

Input preprocessing varied based on model architecture. Images were resized to 224 × 224 pixels for ResNet-50 and MobileNetV2, while YOLO-based models utilized multi-scale inputs ranging from 320 to 640 pixels to improve generalisation.

Cross-entropy loss was used for classification tasks, while segmentation models employed a combination of binary cross-entropy and Dice loss to improve mask accuracy. Model performance was evaluated using standard metrics for both classification and segmentation tasks. For classification models, accuracy, precision, recall, and F1-score were computed, along with confusion matrix analysis to identify misclassification patterns among visually similar dishes.

For segmentation models, Intersection over Union (IoU) and mean Average Precision (mAP) were calculated at IoU thresholds of 0.5 and 0.75 to assess mask quality and boundary precision. These metrics enabled consistent comparison across models and supported the selection of the best-performing architectures for downstream tasks.

### 2.6. Segmentation-Based Portion and Nutrition Estimation

This subsection describes the methodology used to estimate food portions and derive their nutritional profiles from the segmented food images. It covers the segmentation process, portion estimation based on spatial and depth information, and nutritional profiling using the estimated portion and corresponding food composition data.

#### 2.6.1. Segmentation Process

Image segmentation was performed for all food categories using the Roboflow Python SDK (v1.2.11) [29], which provided annotation and dataset management capabilities for preparing segmentation masks. Segmentation annotations were created as pixel-level masks using polygon-based labeling to ensure precise boundary representation of food items. To accommodate dataset size and organizational constraints, the annotation process was distributed across multiple dataset partitions while maintaining a consistent labeling schema across all categories (see annotation protocol in Supplementary Materials on page 4).

Annotated datasets were exported in both YOLO and COCO formats to support compatibility with different segmentation models. The YOLO format was used for training the YOLOv8-based segmentation model, while the COCO format was utilized for training and evaluation with the Mask R-CNN architecture.

Segmentation performance was evaluated using standard metrics including Mean Intersection over Union (mIoU), Dice coefficient, and mean Average Precision (mAP). mIoU measures the average overlap between predicted and ground-truth masks, Dice coefficient evaluates the similarity between two segmentations, and mAP assesses detection and segmentation accuracy across different confidence thresholds.

#### 2.6.2. Portion Estimation Methodology

Accurate portion estimation is a key methodological challenge in image-based dietary assessment, particularly for mixed and visually complex dishes [16]. To address this, a segmentation-driven approach was combined with monocular depth estimation to estimate food volume and mass from a single RGB image without requiring a physical reference object such as a ruler, coin, or printed marker. Instead, the mobile application displays a fixed-size virtual reference box (10 cm × 10 cm) directly on the live camera preview, within which the user positions the food item before image capture. The captured RGB image is processed using the MiDaS monocular depth-estimation model to generate a relative depth map, after which the YOLOv8-Seg food mask is applied to isolate depth information corresponding to the food region. The isolated depth information, together with the virtual reference box as a scale constraint, is then used to estimate food thickness and volume for subsequent mass estimation. Further details of the portion estimation methodology are provided in the Supplementary Materials.

To evaluate the accuracy of the proposed portion estimation method, a validation dataset comprising 200 food images was collected. The ground-truth mass was measured using a digital kitchen scale under controlled conditions.

The estimated mass obtained from the segmentation and depth-based pipeline was compared against the corresponding ground-truth measurements. Quantitative evaluation was performed using standard regression metrics, including Mean Absolute Error (MAE) and Root Mean Square Error (RMSE), defined as:

$$MAE=\frac{1}{N}\sum_{i=1}^{N}\left|M_{i}^{pred}-M_{i}^{true}\right|$$

$$RMSE=\sqrt{\frac{1}{N}\sum_{i=1}^{N}{\left(M_{i}^{pred}-M_{i}^{true}\right)}^{2}}$$

where M_i_^pred^ and M_i_^true^ represent the predicted and actual mass values for the i-th sample, respectively, and N denotes the total number of validation samples.

These metrics provided an objective assessment of the deviation between predicted and actual portion sizes, enabling evaluation of the reliability and practical applicability of the proposed estimation approach.

#### 2.6.3. Nutritional Profiling Methodology

For the nutritional profiling of 83 commonly consumed Pakistani and South Asian dishes, we focused on portion-based estimation of caloric and macronutrient intake, supporting automated dietary assessment and meal planning within the overall system pipeline.

For each dish, a standardized recipe was established based on commonly reported proportions in South Asian culinary literature [30]. Ingredient quantities were normalized to represent a 100 g serving of the finished dish. Nutrient calculations were based on the cooked weight of the final dish. Changes in food weight resulting from moisture gain or loss during cooking were incorporated into recipe standardization procedures to ensure that nutrient values reflected the edible cooked product. Ingredient-level nutritional data were obtained from the USDA FoodData Central database [31]. USDA FoodData Central was selected because of its comprehensive nutrient coverage and standardized food composition information. For ingredients commonly consumed in Pakistan, nutrient values were cross-checked with South Asian food composition references where available to ensure reasonable comparability. Raw ingredient forms were used wherever available, unless only processed alternatives existed (e.g., canned chickpeas). For Pakistani-specific ingredients without direct matches in the USDA database (e.g., ajwain, atta), the closest compositionally equivalent entries were selected.

We acknowledge that nutritional values obtained from the USDA FoodData Central database may not fully represent Pakistani dishes, as their nutritional composition can vary substantially depending on locally available ingredients, cooking methods, recipe formulations, portion preparation, and particularly the quantity and type of oils, spices, and other ingredients used. Where an exact entry for a Pakistani dish was unavailable, the closest compositionally equivalent food entry was selected based on its primary ingredients and general preparation characteristics; however, such equivalence does not guarantee identical nutritional composition, and this is acknowledged as a limitation of the present study.

All cooking components, including oils, ghee, butter, and spices, were incorporated using their corresponding USDA nutritional profiles. Cooking water was excluded from energy calculations but accounted for in nutrient dilution to maintain realistic composition of the final dish.

To ensure the accuracy and reliability of the nutritional profiles, a cross-validation strategy was employed using two established dietary analysis platforms, including ReciPal [32] and FoodWorks Online [33]. ReciPal, a cloud-based recipe analysis system utilizing the USDA database, was used to compute aggregate nutritional values from standardized recipes and generate consistent per-100 g nutrient profiles. FoodWorks Online, a professional dietary analysis software, was used for secondary validation, including detailed micronutrient estimation (e.g., calcium, iron, folate, and vitamin C), assessment of recipe variations (such as the use of ghee versus vegetable oil), and benchmarking against Pakistan-specific recommended dietary allowances [34]. This dual-platform validation approach ensured consistency, improved accuracy, and enhanced the reliability of the nutritional profiles derived across all food categories. However, the present study primarily focused on energy and macronutrient estimation, while micronutrient outputs were generated for validation purposes and will be explored in future work. Details of the methods for nutrient profiling are provided in the Supplementary Materials (see “Portion-Based Nutritional Computation Methodology”).

The estimated nutritional values were qualitatively compared with the corresponding reference values obtained during the validation process. The results showed general consistency for major nutritional components, including calories, protein, carbohydrates, and fat. However, detailed sample-level comparison data were not retained, preventing the retrospective calculation of quantitative agreement metrics. This limitation should be considered when interpreting the nutritional validation results.

### 2.7. Model Integration into Mobile Application

To enable real-time dietary assessment, the trained models were deployed across both server-side and mobile environments, ensuring a balance between computational efficiency and user accessibility.

High-performance models, including ResNet-50 and Mask R-CNN, were deployed on the server side using RESTful APIs implemented with FastAPI [32]. This setup allowed computationally intensive inference tasks to be executed remotely, supporting scalable and efficient processing for web-based and connected mobile applications.

For on-device inference, lightweight models such as MobileNetV2 and YOLOv8n were optimized and exported to TensorFlow Lite (TFLite) [33] and ONNX formats [34]. These models were integrated into the mobile application to enable low-latency predictions without requiring continuous internet connectivity, thereby supporting offline functionality and improving user experience.

A cross-platform mobile application was developed using React Native, integrating modules for food recognition, segmentation, portion estimation, and nutritional analysis. The application provided real-time feedback to users, presenting estimated calorie and macronutrient information based on captured meal images. We also enhanced the system’s adaptability capabilities, which are outlined in the Supplementary Materials (see continuous learning and system evolution).

### 2.8. Usability Testing

To evaluate the usability of the proposed mobile application, a task-based testing approach was employed with users, combined with qualitative feedback and the System Usability Scale (SUS) for quantitative assessment [35,36]. Usability scores were interpreted using SUS score ranges, where above 80.3 is excellent usability and below 51 is poor usability. Details of the methodology are presented in Supplementary Materials (usability testing method).

## 3. Findings

This section presents the findings of the study, covering the development and evaluation of the proposed dietary assessment framework, nutrient profiling, and usability testing. The first subsection presents the dataset preparation, model development and evaluation, including food classification, segmentation, portion estimation, and performance assessment. The subsequent subsections report the nutrient profiling results and the usability evaluation of the developed mobile application.

### 3.1. Dataset Acquisition and Expansion

A total of approximately 20,000 raw images were acquired from three primary sources. Web scraping and YouTube thumbnails, crowdsourcing and external datasets contributed approximately 80% (~16,000), 10% (~2000), and 10% (~2000) of images, respectively. After data cleaning, pixel-level annotations and applying augmentation techniques, the dataset expanded to ~30,000 images, providing a more robust foundation for model training. A category-wise image distribution is given in Table 2.

### 3.2. Model Selection, Training, and Validation

This sub section presents the selection, training, and validation of the deep learning models used in the proposed dietary assessment system. The performance of the classification and segmentation models is first assessed to identify suitable models for food recognition and segmentation. The selected segmentation models are then trained and integrated with the portion-estimation methodology, followed by comprehensive performance evaluation to assess the effectiveness of the overall approach.

#### 3.2.1. Performance of Classification Models

Multiple deep learning models were evaluated, based on classification accuracy. YOLOv8 achieved the highest classification accuracy (~79%), outperforming ResNet-50 (~76%) and MobileNetV2 (~45%). ResNet-50 demonstrated competitive performance but required higher computational resources, making it less suitable for real-time and low resource deployment scenarios. The superior performance of YOLOv8 can be attributed to its ability to capture multi-scale features, which is essential for distinguishing visually diverse and fine-grained food categories.

The classification models were primarily evaluated based on classification accuracy (Table 3). YOLOv8 achieved the highest accuracy of approximately 79%, followed by ResNet-50 at approximately 76%, while MobileNetV2 achieved approximately 45%. The results indicate that YOLOv8 provided the strongest overall classification performance among the evaluated architectures. The comparatively lower performance of MobileNetV2 was observed consistently across our experiments; it was selected primarily as a lightweight model suitable for resource-constrained deployment, and its lower accuracy relative to the heavier architectures can be attributed to the complexity and visual similarity of the food classes, intra-class variation, inter-class similarity, and the limited capacity of a lightweight architecture to learn highly discriminative food features. We have identified this lower MobileNetV2 performance as an explicit limitation of the lightweight classification approach.

#### 3.2.2. Performance of Segmentation Models

To evaluate segmentation performance for portion estimation, multiple instance and semantic segmentation models were assessed on the dataset. Given the real-time and multi-object requirements of the application, both accuracy and computational efficiency were considered in model selection.

#### 3.2.3. Training of Segmentation Models

We first trained Mask R-CNN as a baseline for instance segmentation. After ~85 k iterations, the model learned coarse food categories but struggled with visually similar dishes. We then continued training, which improved overall localization and mask consistency, though fine-grained class errors remained. The final quantitative results (on our test set) were modest as shown in the table. This moderate performance reflects the challenges of our dataset, which has high intra-class similarity and many small items. A breakdown by class highlighted this (Table 4): classes like *jeera_rice*, *ras_malai*, and *white_rice* had high AP (68–74%), whereas under-represented or similar-looking items (*puri*, *kofta*, *beef_karahi*) had very low AP (0–7%). The model also struggled with object size: AP was only 1.27% for small items, 14.55% for medium, and 35.84% for large items, confirming that small-object segmentation is a serious limitation of Mask R-CNN in our context.

We selected YOLOv8-Seg as our primary segmentation model after comparing it with SAM [37], Mask2Former [38], SegFormer (MiT-B2) [39] and a YOLO-based two-stage segmentation pipeline (YOLO26-Seg), as summarized in the Supplementary Materials under Comparison Results. We selected YOLOv8-Seg as our primary segmentation model due to its unified, real-time design. This single-stage model was trained on our pixel-annotated food masks. Qualitatively, YOLOv8-Seg produced clean, accurate masks that closely followed food boundaries, even in crowded plates under varying lighting. It successfully isolated multiple food items in one pass.

Although we did not compute standard metrics (mIoU/Dice) during preliminary model-selection experiments, final evaluation on the designated test set yielded mIoU ≈ 0.6406 and Dice ≈ 0.7810 (Dice and mIoU are mathematically related for the same masks under the standard relationship Dice = 2·IoU/(1 + IoU)).

Preliminary segmentation experiments were conducted during model selection to assess the feasibility of different architectures (SAM, Mask2Former, SegFormer, and the two-stage YOLO26-Seg pipeline); these preliminary experiments did not always use identical evaluation metrics or the same evaluation subset and are therefore reported here as exploratory model comparisons rather than a strictly controlled, head-to-head benchmark. For the final evaluation of the selected model, YOLOv8-Seg was assessed on a designated, held-out test set using standard pixel-level metrics (mIoU, Dice coefficient, and mask AP). Where complete re-evaluation of an earlier model under identical conditions was not possible (e.g., because original prediction masks were not retained), this is explicitly acknowledged as a limitation rather than treated as a directly comparable result.

Table 5 shows YOLOv8-Seg (single-shot, final evaluation) achieved the second-highest mask mIoU among models, behind only the class-agnostic SAM (preliminary evaluation). Mask R-CNN (baseline) had lower overlap but provided strong instance discrimination. Mask2Former lagged in overlap, whereas SegFormer’s metrics rivaled SAM’s. The two-stage YOLOv8 pipeline (YOLO26-Seg) combined detection and segmentation to slightly exceed YOLOv8-Seg’s mask accuracy, though under a preliminary, non-identical evaluation setting.

The confusion matrix results for the YOLOv8-Seg model demonstrate a balanced classification performance, with 78.10% true positives (TP) and approximately 78.00% true negatives (TN) (Table 6). The model produced 21.95% false negatives (FN) and 21.95% false positives (FP), indicating a relatively consistent error rate across positive and negative predictions. Overall, the model achieved a precision of 77.99%, recall of 78.00%, and an F1-score of 78.90%, reflecting a good balance between correctly identifying positive instances and minimizing incorrect predictions.

#### 3.2.4. Portion Estimation

Portion estimation was performed by combining segmentation outputs with pixel-to-weight scaling. The predicted food area was mapped to gram values using calibrated reference portions.

To evaluate performance, predicted portion sizes were compared against manually measured ground truth values for a subset of test samples.

#### 3.2.5. Performance Evaluation

Performance evaluation of the portion estimation module yielded a Mean Absolute Error (MAE) of ±18–25 g and a Root Mean Square Error (RMSE) of 9–12%. Overall, the findings demonstrate that the proposed approach estimates food portions with an average deviation of less than 12% from the ground truth, providing sufficient accuracy for downstream calorie and macronutrient estimation in the nutritional profiling pipeline.

The reported portion-estimation MAE of approximately 18–25 g should be interpreted relative to the size of the food portion. For smaller portions, the same absolute error represents a larger percentage of the actual serving weight, whereas for larger portions its relative impact is reduced. Therefore, absolute MAE alone does not fully characterize portion-estimation performance; the relative error (RMSE of 9–12%) reported alongside the MAE provides a more meaningful, portion-size-independent assessment of accuracy across the tested range of portion sizes.

Although the proposed approach achieves satisfactory portion estimation accuracy, the remaining errors primarily result from irregular food shapes, overlapping food items, variations in food thickness that are not captured in 2D images, and occlusions in plates containing multiple food items. Despite these challenges, the model demonstrates consistent and reliable portion approximation, making it suitable for practical dietary assessment applications. However, from a nutritional perspective, the level of error mentioned above is unlikely to substantially affect estimation of overall dietary patterns. Deviations may be more pronounced for energy-dense foods, where small differences in portion size can result in larger differences in calorie estimation.

We evaluated both a single-stage YOLOv8-Seg model and a two-stage YOLOv8-Classification + Mask R-CNN pipeline (see Supplementary Materials: Combined and Independent Models). While the two-stage approach achieved better classification and segmentation performance, particularly for visually similar food items, YOLOv8-Seg was selected as the primary model due to its faster inference and suitability for real-time applications.

### 3.3. Nutrient Profiling

Following the proposed pipeline, nutritional profiles were generated for 83 dishes across ten food categories.

Clarification on category-level ranges: The calorie and macronutrient ranges reported in Table 7 and Table 8 respectively (e.g., 140–240 kcal/100 g for Meat Curries) represent the variation among different dishes and compositionally equivalent database entries grouped within the same broader food category, for example, the Meat Curries category includes dishes prepared with different meat types, ingredient proportions, and preparation methods, which can result in substantial differences in calories per 100 g. These ranges do not represent measurement uncertainty or prediction error of the proposed system; rather, they illustrate the expected variation in nutritional composition among foods belonging to the same broad category.

The estimated nutritional values generated by the proposed system were compared qualitatively against outputs obtained from ReciPal and FoodWorks, which are widely used nutritional analysis tools. The comparison indicated that the estimated values were generally consistent with reference values for major nutritional components, including calories, protein, carbohydrates, and fat. However, detailed sample-level comparison data were not retained, preventing retrospective calculation of quantitative agreement metrics (e.g., MAE, RMSE, or percentage error). Future work will include systematic nutritional validation using larger datasets and statistical evaluation measures.

### 3.4. Usability Testing Results 

Twenty participants performed usability tasks—capturing a food image, viewing detected items, reviewing nutritional information, and accessing meal analysis results—and completed SUS. Most participants completed tasks successfully with minimal assistance. Overall, participants’ feedback was generally positive, valuing the intuitive interface, speed of processing and obtaining nutritional information from a single food image for dietary monitoring. However, participants felt that the color scheme lacked visual cohesion and did not strongly convey a health/nutrition theme. Moreover, few participants experienced difficulties in framing food correctly within the capture region and navigating between screens. According to the standard SUS interpretation benchmarks the tool was found to be of ‘good usability’, with average SUS score of 78.0 (range 70.0 to 87.5). The findings suggest that the tool is acceptable, but further improvements are recommended for future development and real-world testing.

The detailed participant demographic characteristics (age, gender, technical proficiency, prior dietary-tracking experience, and familiarity with Pakistani cuisine), quantitative task completion rates/times, and the standard deviation/confidence interval for the SUS score were not systematically recorded during this preliminary evaluation.

## 4. Discussion

Automated dietary assessment remains a challenging problem due to the need to accurately recognize mixed food items, estimate portion sizes, and translate these observations into meaningful nutritional information [14,40]. This study addresses these challenges through an integrated machine learning framework specifically developed for Pakistani cuisine. The overall findings indicate that the proposed pipeline can effectively combine these interdependent tasks within a mobile application, demonstrating potential for practical deployment in real-world settings for population health benefits.

The experimental results demonstrate that the proposed framework successfully integrates food recognition, segmentation, portion estimation, and nutrient profiling into a unified dietary assessment system. Among the evaluated classification models, YOLOv8 achieved the best overall performance while maintaining the computational efficiency required for real-time deployment. Its capacity to learn multi-scale visual features [41] proved advantageous for navigating the high intra-class variation and diverse appearance of Pakistani food items. Similarly, YOLOv8-Seg balances mask accuracy and inference speed to reliably extract multiple food regions from complex meal images [42]. While the preliminary comparative evaluation indicated that the two-stage detection–segmentation pipeline (YOLO26-Seg) and class-agnostic models produced marginally higher classification and segmentation accuracy, the single-stage YOLOv8-Seg architecture was ultimately preferred [43]. This choice is justified by its lower computational complexity and faster processing speeds. By using a single-stage approach, the system minimizes the heavy computational overhead and error propagation typical of sequential processing pipelines, making it suitable for resource-constrained, real-time mobile applications, which should be the focus of future research.

The portion estimation results further demonstrate the practicality of the proposed framework. The observed estimation errors remained within an acceptable range for dietary assessment, indicating that the predicted portion sizes are sufficiently accurate for subsequent calorie and nutrient calculations [44]. Most estimation errors can be attributed to inherent challenges associated with monocular image analysis, including irregular food geometry, overlapping food items, occlusion, and the absence of depth information [45]. Nevertheless, these inaccuracies are unlikely to bias overall dietary assessments, as random meal-to-meal errors tend to smooth out when evaluating long-term dietary patterns rather than isolated meal measurements [46]. Collectively, these findings suggest that an efficient, real-time pipeline may offer greater practical value than a marginal increase in predictive accuracy achieved through computationally more demanding architectures.

However, some challenges remain. Firstly, inter-class confusion was observed between visually similar food categories, such as white rice and semolina, reflecting the inherent difficulty of distinguishing foods with comparable color, texture, and appearance. This suggests that incorporating additional contextual or multimodal information could further improve classification performance [47]. Similarly, although the segmentation and portion estimation modules achieved promising results, their performance may decline in more complex scenarios involving overlapping food items, irregular shapes, varying food thickness, and visually similar textures. These findings indicate that aggregate evaluation metrics may not fully capture the challenges encountered in real-world dietary assessment [48].

Future research could also investigate a unified multi-task architecture to jointly execute food classification and segmentation [49]. Integrating these tasks into a single architecture could reduce computational requirements and inference time while minimizing error propagation between sequential processing stages [50]. Such a design has the potential to improve both the efficiency and consistency of the dietary assessment pipeline, particularly for real-time mobile applications where computational resources are limited.

Another critical area for future research is enhancing the depth estimation module. Although monocular depth estimation provides a practical means of estimating food portions without requiring a physical reference object, it inherently predicts relative rather than metric depth. This scale ambiguity reduces estimation accuracy under challenging imaging conditions, such as suboptimal camera positions, lacking distinct visual anchors [41]. Future work should focus on improving the robustness of depth estimation, incorporating real-time feedback to guide users in capturing suitable images, and exploring 3D food reconstruction techniques to enable more accurate volume and portion estimation [44].

## 5. Limitations

Depth and Portion Estimation Limitations: The current system uses monocular depth estimation through MiDaS to estimate relative depth from a single RGB image. Since an independent food-specific ground-truth depth dataset was not available, the depth-estimation component was not evaluated independently. Instead, its effectiveness was assessed as part of the complete portion-estimation pipeline. Consequently, portion-estimation errors may originate from multiple stages, including food segmentation, pixel-to-real-world scaling, depth estimation, volume approximation, and assumptions regarding food density [51]. Future work will incorporate independently measured food height/depth data to evaluate the depth-estimation component separately [52].

Usability Evaluation Limitations: The usability evaluation involved a relatively small sample of 20 participants and was primarily based on the System Usability Scale. Although the mean SUS score of 78.0 indicates a generally positive usability assessment, the study did not systematically retain all participant-level demographic information and detailed task-level performance records. Consequently, the influence of user characteristics, including technical familiarity, prior dietary-tracking experience, and familiarity with Pakistani cuisine, could not be comprehensively analyzed. In addition, individual SUS responses were not linked to the specific model predictions encountered by participants, preventing a statistical investigation of the relationship between perceived usability and technical system performance [53]. Future studies should employ larger and more diverse participant samples and incorporate structured task-completion metrics, participant demographics, individual SUS responses, and user-level system-performance data.

Nutritional Database Representativeness: As noted in Section 2.6.3, USDA FoodData Central values may not fully represent Pakistani dishes prepared with local ingredients and cooking methods; the closest compositionally equivalent entries were used where exact matches were unavailable, and this remains a limitation for the precision of absolute nutrient estimates.

## 6. Conclusions

This study presents an end-to-end system for automated dietary assessment of Pakistani cuisine, bringing together dataset development, food classification, segmentation, portion estimation, and nutrient mapping within a single mobile application. This type of image processing pipeline can offer a practical balance between accuracy and speed for the visually complex, fine-grained food categories found in Pakistani cuisine, and that portion size can be estimated to a reasonable degree of accuracy without specialised hardware. Early usability testing also suggested that the application was manageable for non-expert users, though furthuyjhmner testing and reporting are still needed. Despite challenges related to dataset diversity, visually similar dishes, monocular depth estimation, usability evaluation, and the representativeness of available nutritional databases for Pakistani foods, this work provides a methodological foundation for culturally adaptive, AI-based dietary assessment tools for underrepresented populations. Building on this foundation, future work should focus on independently validating depth-based portion estimation, improving the representativeness of nutritional data, conducting larger and more diverse usability studies, and reducing the processing burden associated with multiple separate models. These improvements would enhance the reliability, scalability, and practical applicability of the proposed system for real-world, everyday dietary assessment.

## Figures and Tables

**Figure 1:**
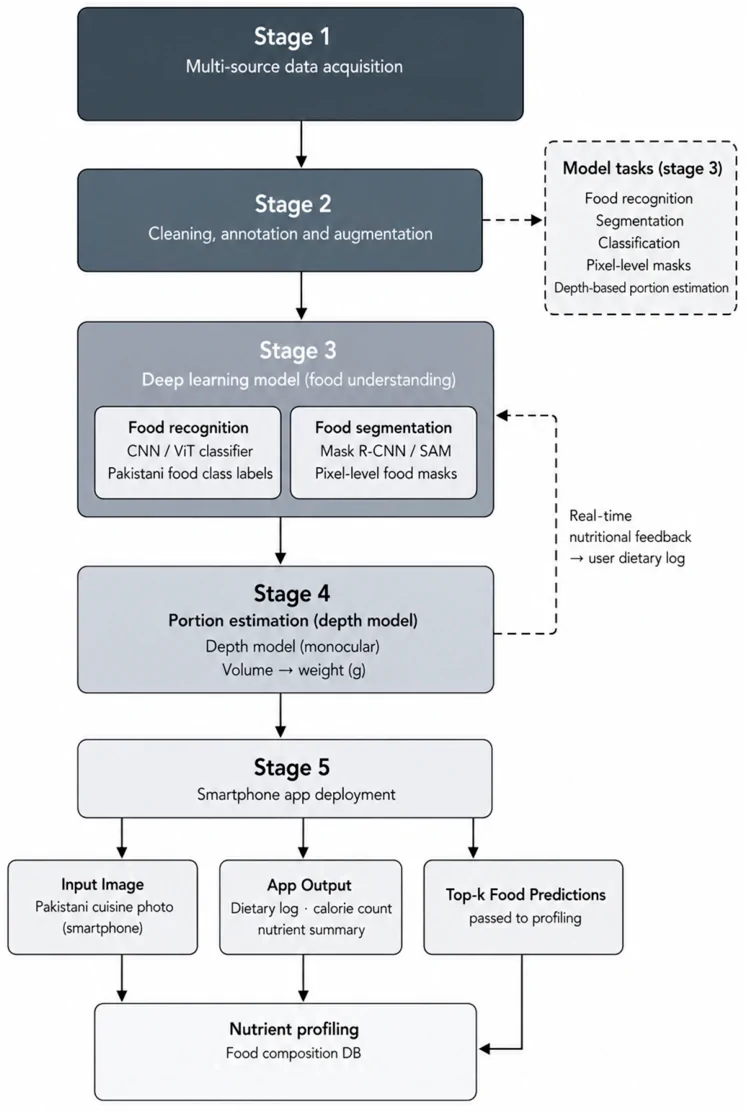
Flow diagram, showing linkages between different stages.

**Table 1:** Dataset Categorization of Pakistani Food Types Used for Model Training.

Category	Examples
Rice Dishes	Biryani, Pulao, Fried Rice, Tahari, Zeera Rice
Meat Curries/Gravies	Karahi, Nihari, Qorma, Haleem, Paya, Kofta Curry
Vegetarian Dishes	Aloo Bhujia, Bhindi Masala, Baingan Bharta, Palak Sabzi
Lentil-Based Dishes (Dals)	Dal Chana, Dal Mash, Dal Gosht, Mixed Dal Fry
Condiments/Sides	Raita, Salad, Kachumar Salad
Breads	Roti, Naan, Paratha, Poori, Sheermal
Snacks & Street Foods	Samosa, Pakora, Gol Gappay, Bun Kebab, Chana Chaat
BBQ/Grills	Chicken Tikka, Malai Boti, Seekh Kebab
Desserts & Sweets	Gulab Jamun, Jalebi, Kheer, Falooda, Shahi Tukray
Beverages	Lemon Sode, Sattu, Soft Drinks, Coffee

**Table 2:** Image distribution across food categories.

Category	Number of Dishes or Classes	Number of Images per Class (Average)	Total Images (Number)
Rice Dishes	13	250	3250
Meat Curries/Gravies	12	250	3000
Vegetarian Dishes	11	250	2750
Lentil-Based Dishes	6	125	750
Condiments/Sides	7	250	1750
Breads	5	250	1250
Snacks & Street Foods	7	250	1750
BBQ/Grills	7	250	1750
Desserts & Sweets	9	250	2250
Beverages	6	250	1500

**Table 3:** Classification Evaluation Metrics (Accuracy, Precision, Recall, F1-score).

Model	Accuracy	Precision (Macro)	Recall (Macro)	F1-Score (Macro)	F1-Score (Weighted)
YOLOv8 Classification	79.0%	~78.5%	~77.8%	~77.9%	~78.8%
ResNet-50	76.0%	~75.5%	~74.6%	~74.8%	~75.7%
MobileNetV2	45.0%	~43.8%	~42.5%	~42.7%	~44.2%

**Table 4:** Baseline Model (Mask R-CNN) Performance.

Metric	Value (%)
Bounding Box mAP	33.47
Segmentation mAP	32.67
AP50	42.87
AP75	35.99

**Table 5:** Segmentation models and their performance.

Models	mIoU	Dice	maskAP	Evaluation Setting	Key Strength
YOLOV8-seg	0.6406	0.4487	0.5433	Final evaluation	Real-time multi-object segmentation
MaskRCNN	0.7540	0.8432	0.3267	Preliminary evaluation	Robust instance-level detail
SAM	0.8752	0.9274	0.7600	Preliminary evaluation	Highly precise boundaries
SegFormer	0.8720	0.9180	0.3193	Preliminary evaluation	Efficient end-to-end segmentation
Mask2Former	0.4511	0.5965	0.2800	Preliminary evaluation	General semantic segmentation
Yolo26-seg	0.6929	0.6658	0.5751	Preliminary evaluation	Combined detection + segmentation

Values are approximate results from our experiments (mIoU/Dice reflect mask overlap; mask AP is reported for YOLO-based models). Mask R-CNN’s mask AP is 32.67% as reported above. The Dice value for YOLOv8-Seg has been corrected to 0.7810 to be internally consistent with its reported mIoU of 0.6406 (see Evaluation Protocol above).

**Table 6:** Confusion matrix of YoloV8-seg.

	Predicted Positive	Predicted Negative
Actual Positive	78.10	21.95
Actual Negative	21.95	≈78.00

**Table 7:** Nutritional values of example dishes (see Supplementary Materials for complete table).

Category	Dish Name	Cal (kcal)	Protein (g)	Carbs (g)	Fat (g)	Na (mg)
Rice Dishes	White Rice	130	2.7	28.2	0.3	1
Meat Curries	Chicken Karahi	200	16	6	13	450
Snacks/Street Food	Samosa	275	5.5	33	13.5	380
BBQ/Grills	Chicken Tikka	185	22	3.5	9	450
Desserts	Gulab Jamun	310	5.5	50	10	80
Breads	Roti	265	8.5	52	3.5	380
Beverages	Chai	45	1	7.5	1	15
Vegetable Dishes	Saag	95	3.2	10.5	5.2	180
Lentils (Daal)	Maash Daal	172	9.1	18.5	7.8	215

**Table 8:** Macronutrients by food category (per 100 g).

Category	Dishes (*n*)	Calories kcal/100 g	Protein g/100 g	Carbohydrates g/100 g	Fat g/100 g
Rice Dishes	13	130–200	2.7–11.5	19.5–28.2	0.3–8.0
Meat Curries	12	140–240	10.0–16.0	4.0–8.0	9.5–17.0
Vegetable Dishes	11	55–115	1.5–3.5	7.0–15.0	2.0–5.0
BBQ/Grills	7	185–280	17.0–22.0	3.0–8.0	9.0–20.0
Breads	5	265–335	7.0–8.5	44.0–52.0	3.5–14.0
Desserts/Sweets	9	145–380	2.5–8.0	20.0–58.0	5.0–15.0
Snacks/Street food	7	140–310	3.5–11.0	14.0–40.0	7.5–16.0
Beverages	6	40–90	0.1–3.0	7.0–13.0	0.5–2.5
Condiments/Sides	7	25–100	0.5–2.5	4.5–8.5	0.3–8.5
Lentils Based Dishes	6	110–120	6.0–7.5	16.0–17.5	2.0–2.5

Note: Calorie ranges represent the variation in nutritional composition among food items within each broad food category and do not indicate prediction uncertainty or estimation error of the proposed system.

## Data Availability

The dataset supporting the findings of this study is publicly available on Kaggle at: https://www.kaggle.com/datasets/alishba7800/smf-dataset (accessed on 15 September 2026). The dataset comprises labeled Pakistani food images across multiple dish classes, organised for food classification tasks. Access requires only a free Kaggle account without any additional permission.

## Supplementary Materials

The following supporting information can be downloaded at: https://www.mdpi.com/article/10.3390/nu18193173/s1 [54,55].
