# Supplementary material for: Developing a Machine Learning–Based System for Nutrient Profiling of Pakistani Cuisine
Source: Nutrients. 2026 Sep 26;18(19):3173. doi: 10.3390/nu18193173 (PMC13667061; doi:10.3390/nu18193173)
Supplement: Supplementary file 1 [file nutrients-18-03173-s001.zip › nutrients-4519868-supplementary.pdf]

## Supplementary Materials

## *Methods*

### Dataset Acquisition

To ensure diversity and represent real-world variability in food presentation, images were collected from the four primary sources:

#### *(a) Web-Based Image Collection*

A substantial portion of the dataset was obtained through web scraping of Pakistani and South Asian food websites and blogs. Automated scripts were used to extract images along with associated metadata, including dish names and source URLs. Data collection adhered to ethical guidelines, including compliance with robots.txt directives and controlled request rates to avoid server overload. Retrieved images were manually reviewed and assigned to predefined categories to ensure labeling accuracy. For instance, dishes such as Sindhi Biryani were categorized under Rice Dishes, while Dal Mash was assigned to Lentil-Based Dishes.

#### *(b) YouTube Thumbnail Extraction*

To capture food images in realistic, non-studio conditions, thumbnails were extracted from popular Pakistani cooking videos. These images reflect authentic household and street-style food presentations, which are often absent in curated datasets. A semi-automated labeling approach was used, where video titles and descriptions were processed using keyword-based matching to assign category labels, followed by manual verification to resolve ambiguities and ensure consistency.

#### (c) *Crowd*-Sourced Data Collection

To further enhance dataset realism, images were collected from voluntary contributors (e.g., researchers and people from their personal networks) using personal smartphone cameras. These food images were captured in natural environments such as homes, restaurants, and cafeterias, without controlled lighting or composition. All participants provided informed consent, and no personal identifiable information was collected. This component improved the model’s ability to generalize to real-world deployment scenarios.

### *(d) Integration of External Datasets*

To address class imbalance and improve representation of underrepresented food categories, additional images were incorporated from publicly available Indian food datasets (Banerjee 2021) with strong visual and semantic similarity to Pakistani cuisine. Approximately 2,000 images were selected and processed using the same cleaning, labeling, and validation pipeline to maintain consistency. These images were used strictly for academic research purposes.

## Portion Estimation Methodology

### (a) Initial Reference-Based Approach

An initial approach utilized a fixed-size physical reference object (10 × 10 cm) placed adjacent to the food item to enable pixel-to-real-world scaling. While this method allowed accurate conversion, it was found to be impractical for real-world use due to the requirement of carrying and positioning a reference object during image capture. Therefore, this approach was not adopted for the final system.

### (b) UI-Based Fixed-Scale Capture with Depth Calibration

To eliminate the need for a physical reference object, a UI-constrained image acquisition strategy was implemented. The mobile application interface defines a fixed-size virtual capture region with known pixel dimensions, ensuring consistent spatial framing across captured images.

Initial attempts to estimate scale using only device-specific parameters (e.g., screen resolution, aspect ratio, and camera field of view) proved unreliable due to hardware variability across smartphones and the absence of dedicated depth sensors. To address this limitation, monocular depth estimation was incorporated into the capture process.

MiDaS (version 3.1) was used to estimate relative depth from a single RGB image (Ranftl et al., 2022). This model was selected due to its ability to generalize across diverse scenes,

computational efficiency, and capability to generate dense depth maps without requiring stereo cameras or specialized hardware.

During image capture, MiDaS computes a relative depth map D(x,y) from the live camera feed. The average depth value within the predefined capture region is calculated to estimate the relative distance between the camera and the food item. Based on empirical experimentation, a valid depth range [$d_{min}$ ,$d_{max}$ ] was defined to ensure stable and consistent scaling. Only images captured within this depth range were accepted for further processing, thereby enforcing both spatial and depth constraints without requiring external reference objects.

### (c) Segmentation and Depth-Based Portion Estimation

Once a valid image was captured, it was processed through a segmentation and depth fusion pipeline to estimate portion size. Firstly, YOLOv8-Seg was used to generate pixel-level masks for each detected food item. For each segmented region, the projected surface area $A_{pixels}$ was computed by counting the number of pixels within the mask. Next, MiDaS provided a corresponding relative depth map D(x,y). Depth values within each segmented region were extracted and averaged to obtain an approximate relative height $h_{relative}$ of the food item.

To convert pixel measurements into real-world units, a scaling factor was derived from the fixed capture region. Let $W_{real\_box}$ and $H_{real\_box}$ denote the known real-world dimensions of the capture region, and $W_{pixel\_box}$ and $H_{pixel\_box}$, their corresponding pixel dimensions. The pixel-to-real-world scaling factor S was computed as:

$S= W_{real\_box} /W_{pixel\_box}$

The real-world surface area was then estimated as:

$A_{real}= A_{pixels} * S^2$

The volume V of the food item was computed by combining the scaled area with the relative height:

$V= A_{real} * h_{relative}$

Finally, the estimated volume was converted to mass using food-specific density values ρ obtained from standardized nutritional databases:

$M = V \times \rho$

This segmentation and depth fusion approach enabled robust portion estimation from a single RGB image while maintaining practical usability across diverse mobile devices.

### (d) Annotation protocol

Pixel-level segmentation masks were created by two annotators, both members of the research team, using the polygon annotation tool provided by the Roboflow platform. During annotation, images were flagged and handled according to specific visual conditions, including horizontal flipping, variable lighting, and blur, to ensure masks remained accurate under these conditions. Formal inter-annotator agreement was not computed for this study, which is acknowledged as a limitation. However, future iterations of the dataset will incorporate a quantitative agreement metric (e.g., Cohen's κ or mean IoU between annotators) on a sampled subset.

## Portion-Based Nutritional Computation Methodology

The system stored nutritional composition values per 100 g for each finished dish and applied linear scaling to estimate nutrient intake for any user-specified portion size. The nutrient content for a given portion was calculated as:

$$N_{portion} = N_{per100g} \times (M / 100)$$

Where:

- **N_portion** = nutrient value for the consumed portion
- **N_per100g** = nutrient content per 100 g of the dish
- **M** = portion size in grams

The computation framework supported multi-dish meal inputs, where individual portion weights were processed independently and aggregated to compute the total nutritional intake for a complete meal. For a meal containing multiple dishes, the total nutrient intake was calculated as:

$$N_{total} = \Sigma N_i \text{ (for } i = 1 \text{ to } k\text{)}$$

Where:

- **$N_{total}$** = total nutrient intake for the meal
- **$N_i$** = nutrient contribution of each individual dish
- **$k$** = number of dishes in the meal

This approach enabled accurate estimation of cumulative caloric and macronutrient consumption across mixed dishes while maintaining consistency with standardized per-100g nutritional references.

**Table 1:** Example of portion-based nutritional computation

| Dish | Portion (g) | Calories (kcal) | Protein (g) | Carbs (g) | Fat (g) | Fiber (g) |
|---|---|---|---|---|---|---|
| Chicken Biryani | 250 | 487.5 | 27.5 | 52.5 | 18.75 | 1.75 |
| Raita | 150 | 82.5 | 3.75 | 8.25 | 3.75 | 0.45 |
| **TOTAL MEAL** | **400** | **570.0** | **31.25** | **60.75** | **22.5** | **2.20** |

Table 1 illustrates an example of portion-based nutritional computation for a mixed meal consisting of Chicken Biryani (250 g) and Raita (150 g). Nutritional values for each dish were calculated using the per-100g composition and scaled according to the specified portion sizes. The total nutritional intake for the meal was obtained by summing the individual contributions of each dish, demonstrating the system’s ability to handle multi-item meals and provide aggregated dietary information.

## Continuous Learning and System Evolution

To ensure sustained performance improvement and adaptability to diverse food categories, the system incorporated a feedback-driven continuous learning framework.

User feedback was collected through manual corrections to model predictions, which were logged and incorporated into the training dataset. This enabled the system to learn from real-world usage and correct misclassifications over time. An active learning strategy was employed to prioritize ambiguous or underrepresented samples. Data instances associated with high model uncertainty were identified and selected for manual re-annotation, improving the quality and representativeness of the dataset. The dataset was further expanded by gradually incorporating regional and cross-cultural dishes, enhancing coverage and enabling the model to generalize across a wider range of food types. Using this approach, model performance was continuously refined through periodic retraining cycles, where newly collected and annotated data were integrated into the training pipeline. This iterative process ensured ongoing improvement in accuracy, robustness, and generalization capability.

## Usability testing method

### (a) Participant recruitment and testing procedure

A total of 20 undergraduate students were recruited from the university, irrespective of their academic discipline. All participants were regular smartphone users. Informed consent was obtained prior to their participation in the study. Three usability testing sessions were conducted on campus, each lasting approximately 20 minutes. During each session, participants were asked to complete a set of predefined tasks using the mobile application (see table 3). After completing each task, participants provided qualitative feedback by responding to the following open-ended question:

> *"What difficulty did you experience in completing the task, and how can it be improved?"*

At the conclusion of all tasks, participants completed a paper-based System Usability Scale (SUS) questionnaire in their preferred language (English or Urdu), enabling standardized evaluation of perceived usability.

**Table 3: Usability testing tasks**

| Task | Instructions to complete task |
|---|---|
| Capture meal image | Open the application; position the food within the fixed-size capture region; capture the image. |
| Review nutrition | After image capture, view the estimated calorie and macronutrient breakdown (protein, carbohydrates, fat). |
| View summary | Navigate the meal history screen and review the aggregated nutritional summary for the session. |

## Findings

| Category | Dish Name | Cal (kcal) | Protein (g) | Carbs (g) | Fat (g) | Na (mg) |
|---|---|---|---|---|---|---|
| Rice Dishes | White Rice | 130 | 2.7 | 28.2 | 0.3 | 1 |
| | Chicken Biryani | 195 | 11.0 | 21.0 | 7.5 | 380 |
| | Beef Biryani | 200 | 11.5 | 20.5 | 8.0 | 400 |
| | Mutton Biryani | 200 | 11.0 | 21.0 | 8.0 | 400 |
| | Jeera Rice | 155 | 2.8 | 28.5 | 3.2 | 210 |
| | Khichdi | 110 | 4.2 | 18.5 | 2.5 | 180 |
| | Matar Rice | 148 | 3.2 | 26.5 | 3.1 | 165 |
| | Chicken Pulao | 168 | 9.5 | 22.1 | 4.6 | 245 |
| | Egg Fried Rice | 140 | 4.0 | 19.4 | 5.1 | 190 |
| | Mutton Pulao | 260 | 18.0 | 22.0 | 12.0 | 300 |
| Meat Curries | Chicken Karahi | 200 | 16.0 | 6.0 | 13.0 | 450 |

| | | | | | | |
|---|---|---|---|---|---|---|
| | Beef Karahi | 210 | 15.5 | 5.5 | 14.0 | 470 |
| | Nihari | 220 | 14.0 | 6.0 | 15.5 | 480 |
| | Chicken Korma | 230 | 14.0 | 7.0 | 16.5 | 420 |
| | Fish curry | 145 | 14.5 | 5.5 | 7.8 | 310 |
| | Paye | 180 | 10.5 | 4.0 | 13.5 | 420 |
| | Keema | 190 | 12.5 | 6.5 | 13.8 | 380 |
| Snacks / Street | Haleem | 165 | 11.0 | 16.0 | 6.5 | 420 |
| | Samosa | 275 | 5.5 | 33.0 | 13.5 | 380 |
| | Fries | 310 | 3.3 | 39.5 | 15.0 | 210 |
| | Gol Gappy | 132 | 3.6 | 23.0 | 3.5 | 215 |
| | Pakode | 310 | 7.0 | 28.0 | 16.0 | 600 |
| BBQ / Grills | Chicken Tikka | 185 | 22.0 | 3.5 | 9.0 | 450 |
| | Seekh Kebab | 230 | 18.5 | 4.5 | 15.5 | 430 |
| | Chapli Kebab | 280 | 17.0 | 8.0 | 20.0 | 480 |
| | Malai Boti | 220 | 18.0 | 5.0 | 14.0 | 600 |
| Desserts | Gulab Jamun | 310 | 5.5 | 50.0 | 10.0 | 80 |
| | Kheer | 145 | 3.5 | 22.0 | 5.0 | 55 |
| | Zarda | 325 | 4.5 | 50.0 | 11.5 | 13 |
| | Falooda | 185 | 3.0 | 30.0 | 6.0 | 50 |
| | Kulfi | 253 | 6.0 | 26.0 | 14.0 | 40 |
| | Barfi | 393 | 12.5 | 28.2 | 25.5 | 33 |
| | Jalebi | 317 | 3.4 | 44.6 | 13.8 | 67 |
| | Suji Halwa | 315 | 3.9 | 40.6 | 15.3 | 15 |

| | | | | | | |
|---|---|---|---|---|---|---|
| | Gajar Halwa | 276 | 4.5 | 28.1 | 16.1 | 29 |
| Breads | Roti | 265 | 8.5 | 52.0 | 3.5 | 380 |
| | Naan | 280 | 8.0 | 50.0 | 5.5 | 430 |
| | Paratha | 290 | 7.5 | 44.0 | 9.5 | 350 |
| | Puri | 332 | 6.5 | 39.4 | 16.5 | 192 |
| | Anda Paratha | 280 | 10.0 | 30.0 | 15.0 | 400 |
| Beverages | Chai | 45 | 1.0 | 7.5 | 1.0 | 15 |
| | Coffee | 45 | 1.2 | 6.8 | 1.5 | 18 |
| | Lassi | 92 | 3.1 | 12.8 | 3.2 | 42 |
| | Sattu | 350 | 12.0 | 60.0 | 2.0 | 6 |
| | Lemonade | 50 | 0.0 | 12.0 | 0.0 | 200 |
| | Milk Soda | 60 | 1.0 | 10.0 | 0.0 | 60 |
| Vegetable Dishes | Saag | 95 | 3.2 | 10.5 | 5.2 | 180 |
| | Bhindi | 85 | 2.0 | 9.5 | 4.8 | 160 |
| | Aloo Baingan | 100 | 2.5 | 10.0 | 6.0 | 400 |
| | Palak Gosht | 150 | 12.0 | 6.0 | 10.0 | 475 |
| | Ghiya | 20 | 1.0 | 4.0 | 0.2 | 300 |
| | Karela | 25 | 1.0 | 5.0 | 0.3 | 350 |
| | Aloo Methi | 120 | 5.0 | 15.0 | 8.0 | 400 |
| | Mixed Sabzi | 90 | 3.0 | 12.0 | 6.0 | 350 |
| | Aloo Gobi | 110 | 4.0 | 12.0 | 10.0 | 300 |
| | Aloo Palak | 120 | 4.0 | 14.0 | 8.0 | 350 |

| | | | | | | |
|---|---|---|---|---|---|---|
| Lentils (Daal) | Maash Daal | 172 | 9.1 | 18.5 | 7.8 | 215 |
| | Mixed Daal | 116 | 7.2 | 14.8 | 3.2 | 185 |
| | Chana Daal | 130 | 8.2 | 16.5 | 3.8 | 195 |

*Source: USDA FoodData Central; validated via ReciPal and FoodWorks Online.*

This nutritional table is directly integrated into the pipeline, where segmentation estimates food portion size and the per-100g values from the table are scaled accordingly to compute final nutrient intake. This enables image-based food detection to be translated into accurate nutrition values for Pakistani foods in real time.

# 3.8 Validation and Accuracy Assessment

To ensure reliability, a multi-stage validation strategy was applied.

### 1. Cross-Tool Validation

Nutritional values were compared across three sources:

- USDA FoodData Central
- ReciPal
- FoodWorks Online

Evaluation was performed on **15 randomly selected dishes**.

| **Metric** | **Value** |
|---|---|
| Mean Deviation | **8.4%** |

| | |
|---|---|
| Standard Deviation | **±2.1%** |

This variation falls within expected bounds due to differences in ingredient composition and cooking methods.

**Justification of Sample Size:**
The subset of 15 dishes was selected to ensure representation across all major food categories while maintaining feasibility for manual verification.

## 2. Literature Benchmarking

Generated values were compared with established South Asian food composition references (e.g., IFCT 2017).

- Macronutrient deviation remained within **±10%**, confirming consistency with published benchmarks.

## 3. Portion Scaling Verification

The scaling mechanism was validated across multiple portion sizes (50g–500g) for 10 dishes.

- Results confirmed **linear proportionality**
- No rounding inconsistencies were observed

## Comparison results to select the best segmentation models

We also evaluated several state-of-the-art segmentation methods on our data for comparison:

- **SAM (Segment Anything Model):** Achieved *very high* mask overlap: **mIoU = 0.8752, Dice = 0.9274. SAM generated >30k masks** with excellent boundary adherence. This shows SAM's

power at fine-grained segmentation. However, SAM is class-agnostic (no built-in classification), so its practical value for multi-class food segmentation is limited without additional labeling.

- **Mask2Former:** Achieved moderate performance: **mIoU = 0.4511, Dice = 0.5965, Pixel Accuracy ≈ 0.8268**. It captured coarse food regions but often missed exact boundaries. These scores indicate that Mask2Former does not align as well with our ground-truth masks in this task. Its strength is general semantic segmentation, but it underperforms on precision compared to our baseline.
- **SegFormer (MiT-B2 variant):** Showed strong semantic segmentation: **pixel Accuracy ≈ 0.89, mIoU = 0.872, Dice = 0.918**. In practice, SegFormer's masks were smooth and class-coherent. It does end-to-end segmentation without separate detector. However, on our multi-object food scenes SegFormer's performance fell slightly below Mask R-CNN in terms of instance-level detail; for example, some overlapping foods could be merged.
- **"YOLO26-Seg:** This refers to using YOLOv8 for detection/classification followed by mask prediction. It achieved **mIoU = 0.6929, Dice = 0.6658, and mask mAP ≈ 0.5751**. This hybrid approach produced accurate masks (good overlap with GT) and benefited from a strong classifier. Its performance was slightly better than our single-shot YOLOv8-Seg mask accuracy but requires twice the model (detector+mask).

# Combined and independent models

## 3.6 Pipeline Comparison: Single-Stage vs. Two-Stage Approach

Table  summarises the comparative analysis of the two main segmentation pipelines evaluated in this study.

***Table 6:.*** *Comparison of single-stage and two-stage segmentation pipelines.*

| **Pipeline** | **Description** | **Strengths** | **Limitations** |
|---|---|---|---|
| **YOLOv8-seg** | End-to-end model predicting class and | Accurate masks in most cases; simpler setup; faster inference | Classification accuracy is low, especially for similar food items; mask may slightly over/under- |

| | | | |
|---|---|---|---|
| | instance masks in a single step | | segment small or thin objects |
| **YOLOv8-class + Mask R-CNN** | Two-model pipeline: YOLOv8 performs classification → Mask R-CNN segments the object | High classification accuracy; highly accurate masks; flexible pipeline | Slower inference; requires more GPU memory; more complex to deploy |

Qualitative comparison confirms that the two-stage pipeline correctly classifies visually challenging items (e.g., French fries vs. meethee chawal) where the single-stage YOLOv8-Seg may assign incorrect class labels with low confidence. For deployment contexts where latency constraints are paramount, YOLOv8-Seg remains the recommended primary model.

**YOLOV8 - SEG** **YOLOV8 CLASS + MASK RCNN**

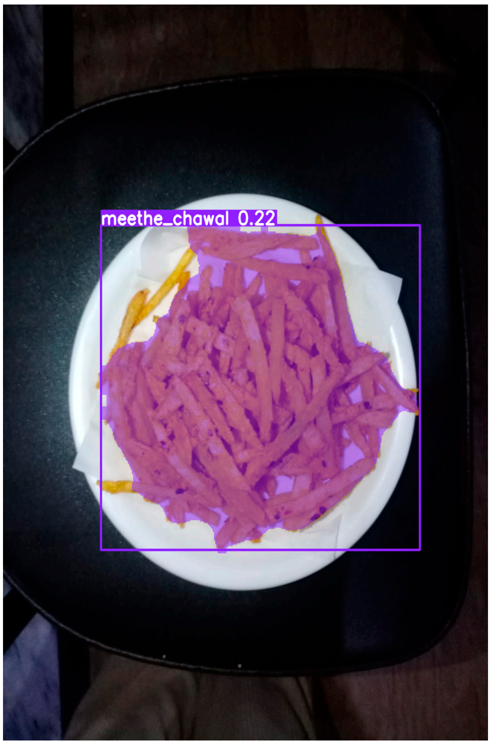


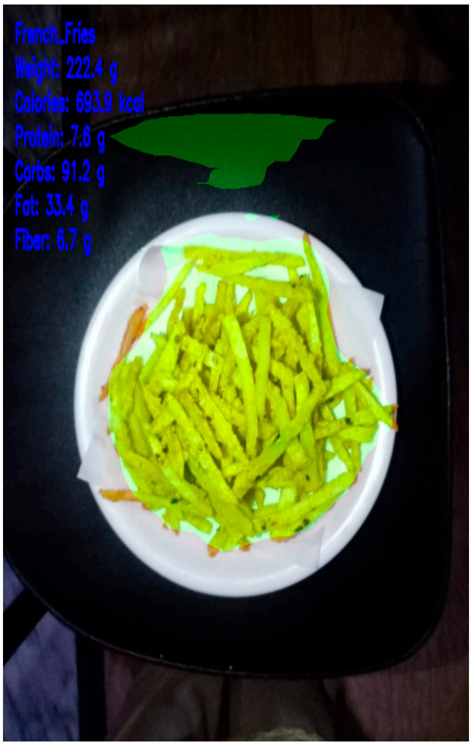

## *References*


Ranftl, R., Lasinger, K., Hafner, D., Schindler, K., & Koltun, V. (2022). Towards Robust Monocular Depth Estimation: Mixing Datasets for Zero-Shot Cross-Dataset Transfer. *IEEE Transactions on Pattern Analysis and Machine Intelligence*, *44*(3), 1623–1637. https://doi.org/10.1109/TPAMI.2020.3019967

Banerjee, S. (2021). Indian Food Images Dataset [Dataset]. Kaggle. https://www.kaggle.com/datasets/iamsouravbanerjee/indian-food-images-dataset
